# Facial Paralysis

**Published:** 1882-03

**Authors:** Jas. T. Whittaker

**Affiliations:** Prof. Theory and Practice of Medicine, Medical College of Ohio


### Independent Practitioner.

VOL. III.——*March*, 1882.——NO. 3.

### CLINICAL LECTURES.

*We are not responsible for the opinions expressed by contributors.*

ARTICLE I.

### FACIAL PARALYSIS.

### GOOD SAMARITAN HOSPITAL, CINCINNATI, O.

JAS. T. WHITTAKER, M. D., Prof. Theory and Practice of Medicine, Medical College of Ohio.
Reported by J. M. FRENCH, M. D., Assistant to the Chair of Practice.

GENTLEMEN :—

Here is an individual with a physiognomy so distinctive of his disease as to name it. We may say of it that it is written across his face in plain, unmistakable characters. You will very seldom see the disease so well marked as this. Upon the right side you can see the folds on the forehead perfectly distinct, but upon the left side the skin is perfectly smooth. You will here appreciate the remark that Romberg once made "*Fuer alte Frauen, k[illegible]n besserer kosmetik existirt,*" "For old women there is no better cosmetic." It completely effaces all wrinkles. I need hardly mention to you the name of this affection—facial paralysis, of the kind we most frequently see, peripheral. We sometimes, though rarely, see a paralysis in which both sides of the face are affected, a bilateral but painless affection. You can hardly have a more pitiful picture presented than an individual thus affected.

He looks as if he wore a mask. If I were to hold a piece of card board up in front of this man's face and let you look at the two sides separately, you would say: These are the faces of different men.

The most pronounced of all the changes concern the eye. This man cannot close his left eye. He has paralysis of the orbicularis palpebrarum. His eye does not close because there is this paralysis of the orbicularis palpebrarum, but it is held open because of the persistent action of the levator palpebræ superioris, which being supplied by a branch of the third nerve, is not implicated in the disease. The orbicularis, you know, is supplied by the facial and is the muscle which, as a general rule, suffers most in peripheral paralysis.

This disease usually comes on suddenly, frequently in the night. Sometimes the patient does not notice it until his attention is called to the deformity by his friends. We had an individual here last year who did not notice anything wrong until some one told her she "stared in her sleep." Now, the more complete the paralysis, the better is the prognosis, strange as it may sound. When an individual comes to you with his eye wide open, staring, the prognosis is good, but when he can shut his eye it is bad. This sounds paradoxical, but it is nevertheless true, as a rule, because when the eye is open, it shows that all the fibres of the muscle are paralyzed, in other words the paralysis is peripheral and not central. If an individual have a lesion in the brain, he very frequently has a facial paralysis, you know, but with a central lesion, the individual is nearly always able to close his eye, because the central lesion depends upon a blood-clot or plug in an artery which affects the origin of the nerve. But you can understand from the numerous fibres of origin of the facial nerve how a central lesion could hardly possibly affect all the fibres, so that an individual with a central lesion can open his eye, whereas, an individual with a peripheral lesion somewhere in the course or distribution of the trunk nerves has his eye open. This is a very important point, for first of all we want to know when a man comes to us with a facial paralysis, whether it is due to a lesion of the brain, or is simply an affection of the surface. Then again, when we see a facial paralysis due to a central lesion it is nearly always in a hemiplegic patient, but the individual may have recovered from his other paralysis, the facial paralysis being the last to disappear. But an individual may have a facial paralysis with no other paralysis and still the lesion be in the brain, so that the condition of the orbicularis is the first point to fix.

Now, we know of this individual that he never had any paralysis of the arm or leg, and we see the staring eye, consequently we know that the lesion is not centric. The lesion is outside the brain; we have to deal with a peripheral paralysis, the form which used to be known as Bell's paralysis. We have now then to determine what caused the paralysis, second where its cause is situated, and third its treatment; but look at the patient first of all. We know that, if this paralysis of the ring muscle of the eye continues for some time, the patient will have perhaps serious damage ensue from dust and coal-soot lodging upon the surface of the eye, but he can always prevent this danger, not by a voluntary movement of course, but by occasionaly closing the eye by artificial means, and I have more than once seen full protection given, by simply adjusting a piece of adhesive plaster over the lids to keep them shut. Our patient has lost the power of all the muscles of his face, and hence he cannot laugh or smile, he cannot whistle, he cannot express himself in his face. He can eat, he has full control over the movement of his jaws, but he cannot masticate his food as well as ever, he cannot keep the alimentary bolus, as the physiologists say, between his teeth, because of paralysis of the buccinator and so he must help himself by putting his fingers into his mouth. Therefore these patients are not so helpless as those whose fifth nerve is paralyzed, as from the abolition of sensation particles of food accumulate between the cheek and jaw until they become rancid and decomposed. Our patient can chew because the muscles of mastication, the temporal, masseter, pterygoids, being supplied by the inferior maxillary branch of the trifacial nerve are not affected in this paralysis.

In the history we have gotten from our patient we learn that he was attacked about ten weeks ago with a severe pain under the left ear, which continued three weeks, and to an application made to relieve this pain, he attributes his paralysis. You know that there are recurrent sensory fibres running back from the fifth nerve to the facial, like those from the posterior to the anterior spinal nerves, so that sensations are perceived in the course of the facial, a purely motor nerve.

In old times the surgeons used to cut and cut out the facial nerve in relief of facial neuralgia. Now, however, we know that the facial is at its origin exclusively a nerve of motion, and that it has nothing to do with conducting the sense of pain. This man's pain, then, was due to an affection of the fifth nerve from the same cause probably, which paralyzed the facials. After his pain had continued for some time he rubbed his face with a liniment, and the next morning he got up with his face dragged to one side. Naturally enough from the force of the *post hoc* argument, he attributes the paralysis to the liniment, but it could have had, of course, nothing to do with it. The pain was the commencement of the trouble, for when the pain subsided, the paralysis came on ; he went to sleep with the pain, and awoke with the paralysis. Patients frequently come to you in distress and dismay on account of this paralysis. It comes on mostly suddenly. It comes on, however, nearly always after some exposure to cold which the patient can remember. A man gets it from riding in a car near an open window ; or an individual sleeps beside a window that is open, or near a door, or in a draught that comes through a crevice. This man does not remember any exposure. In the vast majority of cases, however, the disease comes on after an exposure which the individual has recognized as the cause. But then cold is not the only cause of it. It might be the only cause of it if the lesion affected only the surface or the extreme periphery of the nerve, but anything will produce it which will affect the nerve anywhere in its course. To locate the lesion we must study the anatomy of the nerve.

The facial nerve emerges at the lower border of the pons varolii, near the medulla oblongata. From this origin a large membrane of fibres may be traced backwards into the pons varolii, where on the border of the floor of the fourth ventricle it connects with the same nucleus as the eighth or portio mollis nerve. It is also connected with this nerve by a small fasciculus, the portio inter duram et mollem. The nerve then passes forwards upon the crus cerebri to the auditory meatus. Entering this it passes first upon the inner side and then upon a groove in the auditory nerve. It then passes into the aqueductus Fallopii and follows its tortuous course throughout to its exit at the stylo-mastoid foramen. But in this aqueductus it gives off three branches, the superficial, internal and external petrosal ; and that singular nerve, the chorda tympani, of which it used to be said that it comes from a nerve of sensation, joins a nerve of motion and becomes a nerve of special sense. By the superficial petrosal it is connected with Meckel's ganglion, to which it supplies its motor root. By the internal it communicates with the otic ganglion, and the external branch unites it with the sympathetic filaments accompanying the middle meningeal artery; leaving the canal at the stylo-mastoid foramen it becomes superficial to form the well-known pes anserinus. Now, by remembering these communications, you can distinguish the point at which the lesion has occurred.

The two branches, the greater and less, or the superficial and internal petrosal go to ganglia, the former to Meckel's, the latter to the otic. From the otic, branches pass to the tensor palati and tensor tympani muscles. As it passes out of the stylo-mastoid foramen it communicates with the pneumogastric, the sympathetic and glossopharyngeal nerves. The superficial petrosal supplies, through Meckel's ganglion, the palate, the levator palati and azygos uvulæ. There is one more nerve we have to consider, and that is the chorda tympani. This is one of the most curious nerves in the body. Just where the chorda tympani arises is still a matter of dispute. It was once thought to be a nerve which arose with the tri-facial and as it passed out in the same sheath with it, gradually became a nerve of special sense and separated from it to be distributed to the tongue, supplying it with the sense of taste. Now, however, we know that it has nothing to do with the sense of taste directly; its office being to regulate the supply of blood to the tongue and the secretion of the submaxillary gland, thus governing the erection of the papillæ, and influencing the sense of taste only to the degree to which these functions are associated with it. It carries an impulse out to the tongue, not from it. The submaxillary gland is sometimes said to be the gland of taste. When the mouth waters, the flow comes largely from this gland and a sudden stimulus of taste is felt as painful distension under the jaws. In locating the seat of the lesion in a case of facial paralysis, you will look first into the throat and see whether the palate is deflected. If you find a deviation of the palate, one side being paralyzed, you know that the lesion has occurred previous to the giving off of the superficial petrosal nerve. In this man, however, the uvula is perfectly natural and straight, and we know from this fact that the paralysis is located on this side of the great petrosal. As I hold my watch to his ear he can hear the ticking only a very short distance from it, but at about the same distance on both sides. Now, if the lesion were back of the smaller petrosal branch, the tensor tympani muscle would be paralyzed, and the laxator muscle making the membrane less tense, the hearing would be more acute. Individuals with the lesion located back of this nerve come to us sometimes with a supernatural sense of hearing. The disease then in this case, the hearing not being affected, is on this side of that particular point. Let us go a little further: the chorda tympani ministers indirectly, as we have seen to the sense of taste; that is, it regulates the secretion of saliva in the submaxillary gland, and it controls the circulation in the papillæ of the tongue, putting them into a position ready to receive the impression of taste. The sense of taste on this left side of the tongue ought then to be diminished, but it is not, for the patient tastes as well on one side as the other. These individuals seldom notice any difference in their ability to taste on one or the other side before coming to you at all; so you may test it for yourself. A little salt is about the best substance for testing this condition.

Now, if we had found in this case no affection of the palate or of the hearing, and yet had found an affection of the sense of taste, we could have said that this lesion was situated between the giving off of the petrosal branches and the chorda tympani; but as the taste is unimpaired, we must conclude that the lesion is still more superficial.

With just such nicety can you diagnosticate the location of lesions of this nerve.

Now, to what cause can this paralysis be due? It might be due, say, in general, to a periostitis, to syphilis, or a chronic disease of the ear. This individual has had an occasional discharge from his ear, which he says, came on once in six months or perhaps once a year, but he describes the discharge as a clear, watery fluid. I do not think that is evidence enough to justify us in saying that there is any caries here.

Anyone, from a light aural catarrh, might have an occasional discharge of fluid from the ear, and yet not have any affection of the bone.

Further, the uvula hangs perf[illegible]ly straight, he has no affection of the sense of hearing or taste, and from all these factors we can say that the disease is situated entirely on the outside and is purely a case of peripheral paralysis. It is not in the aqueductus Fallopii, nor in the auditory meatus, but wholly external. This is the most common kind of paralysis we have to treat.

The prognosis is more and more favorable as you bring the lesion out further toward the periphery ; and when this is situated entirely in the superficies of the nerve the prognosis is most favorable. It depends for the most part upon the age of the disease. This man can speak pretty well, but of course he has difficulty with the labials.

I know of no disease with which you could confound peripheral paralysis. In the case I showed you last week we made the diagnosis of dementia paralytica, from the falling away, among other signs, of the lip from the lower jaw. But you could not confound such a case as this with one of progressive paralysis of the insane, for there is here no insanity.

Now, what is the end of a case like this? In a case like this, the prognosis is perfectly good. This is the kind of paralysis that a man gets from pressure upon a nerve. He goes to sleep with his arm hanging over the back of a chair and awakes with the arm paralyzed. Sometimes we have this paralysis in the newly-born child, where the forceps have been applied. The prognosis in these cases is always good, the paralysis soon passing off. I saw a case once where the paralysis lasted nearly two months; then the child recovered and completely regained control of the muscles of the face. There is not a case on record, so far as I could see, of paralysis in the newly-born that did not recover where the paralysis was due to pressure by the forceps.

Now the same means that enables you to cure this disease enables you to make the prognosis. We have here wires running from two batteries, the galvanic and the faradic. If we can get up contraction of the muscles that are affected, with either of these currents, the prognosis is completely favorable; if with the faradic, more so than with the galvanic.

Now see what a mistake you might make with only a casual observation. You have for instance, a case of central lesion ; you apply the battery, and of course get a reaction. You make a favorable prognosis, and are deceived with the result. But I am now speaking only of peripheral paralysis. When in them you secure a reaction, you can assure the patient that he will ultimately get well. Suppose you failed with the smaller faradic battery, would you then give up the case? Not at all. You must then try the constant or galvanic current; the interruptions being produced either by removing one of the poles from the surface so as to break the current in that way, or by means of a clock-work or rheotome connected with the battery.

Now we did not know for a long time the explanation of this difference in the action of the two currents. The old clinicians used to try the faradic current, and if there was no response, the case was dismissed. But now we know that cases will respond to the galvanic current after failure to the induced current, because of the longer time the current is allowed to act upon the muscles, the constant current—using the word "constant" as synonymous with galvanic—acting longer than the faradic. Now just so long as the reaction in these degenerated muscular and nervous fibres exists, there is hope for the patient. You can cultivate the tone of the muscle from the faintest fibrillation up to good contraction. I have seen a good many cases that had been abandoned by physicians, because they could not get any contraction from the faradic current. I have had patients come to me with this disease, who, when I was about to apply the battery would say, they had "tried electricity and it had failed," where the faradic current did fail, but where the galvanic produced excellent results. The time of an application should not exceed ten minutes, or you will exhaust the irritability of the muscle and nerve. Now, as I apply the faradic current, placing one pole back of the ear and passing the other pole over the peripheral distribution of the nerve, I cause (as you see) a contraction of the muscles of the neck so that his whole head is moved, but I get no motion of the muscles of the face. There is no response whatever to faradic electricity. Now let us try the interrupted galvanic current. Here you see we get good reaction at once, and hence we can assure our patient that he will probably ultimately recover.

We say as a rule concerning the strength of the application of the current, that it should be perceptible but not painful. Whatever causes pain does harm, so the application should be distinctly felt, but should not give rise to pain. The truth is an effective current causes rather pleasurable than painful sensations. Just so long as you can get up the faintest contractions with any kind of current, the case is full of hope; for you can cultivate, I say, the finest muscular fibrillar contraction into a full restoration of contractility in time, so that the face will again resume its own expression, and the individual can speak with distinctness. Now we will use this interrupted galvanic current on this man every day, then, after awhile, as soon as we can produce contractions with the faradic, we will use it. When that time comes, the rest of the case is easy enough.

